# The Effect of Psilocin on Memory Acquisition, Retrieval, and Consolidation in the Rat

**DOI:** 10.3389/fnbeh.2014.00180

**Published:** 2014-05-16

**Authors:** Lukas Rambousek, Tomas Palenicek, Karel Vales, Ales Stuchlik

**Affiliations:** ^1^Institute of Physiology, Academy of Sciences of the Czech Republic, Prague, Czech Republic; ^2^Prague Psychiatric Center, Prague, Czech Republic

**Keywords:** psilocin, spatial memory, Carousel maze, Morris water maze, allocentric navigation, hallucinogenic alkaloids, learning, memory

## Abstract

The involvement of the serotonin system in the pathophysiology of schizophrenia has been elucidated by experiments with hallucinogens. Application of a hallucinogen to humans leads to changes in perception, cognition, emotions, and induction of psychotic-like symptoms that resemble symptoms of schizophrenia. In rodent studies, their acute administration affects sensorimotor gating, locomotor activity, social behavior, and cognition including working memory, the phenotypes are considered as an animal model of schizophrenia. The complexity and singularity of human cognition raises questions about the validity of animal models utilizing agonists of 5-HT_2A_ receptors. The present study thus investigated the effect of psilocin on memory acquisition, reinforced retrieval, and memory consolidation in rats. Psilocin is a main metabolite of psilocybin acting as an agonist at 5-HT_2A_ receptors with a contribution of 5-HT_2C_ and 5-HT_1A_ receptors. First, we tested the effect of psilocin on the acquisition of a Carousel maze, a spatial task requiring navigation using distal cues, attention, and cognitive coordination. Psilocin significantly impaired the acquisition of the Carousel maze at both doses (1 and 4 mg/kg). The higher dose of psilocin blocked the learning processes even in an additional session when the rats received only saline. Next, we examined the effect of psilocin on reinforced retrieval and consolidation in the Morris water maze (MWM). The dose of 4 mg/kg disrupted reinforced retrieval in the MWM. However, the application of a lower dose was without any significant effect. Finally, neither the low nor high dose of psilocin injected post-training caused a deficit in memory consolidation in the MWM. Taken together, the psilocin dose dependently impaired the acquisition of the Carousel maze and reinforced retrieval in MWM; however, it had no effect on memory consolidation.

## Introduction

Psilocybin (*O*-phosphoryl-4-hydroxy-*N,N*-dimethyltryptamine) and its active metabolite psilocin (4-hydroxy-*N,N*-dimethyltrypt amine) are the main psychoactive indolealkylamines contained in hallucinogenic mushrooms (Hasler et al., 1997; Passie et al., 2002; Tyls et al., 2013). They act as agonists at serotonin receptors, mainly 5-HT_2A/C_ and 5-HT_1A_ subtypes (for a review, see Tyls et al., 2013). Recently, affinities to other than serotonergic receptors have also been reported (e.g., D1 and D3 receptors); however, their role in the neurobiology of psilocin’s and psilocybin’s action is disputable (Ray, 2010). Nevertheless, most of the evidence agrees that hallucinogenic effects are mediated mainly via agonism at postsynaptic 5-HT_2A_ receptors with a contribution of 5-HT_2C_ and 5-HT_1A_ receptors (Nichols, 2004). A secondary role of the activated limbic dopaminergic system also seems to be responsible for some of its effects (Vollenweider et al., 1999).

The acute effects of psilocybin in humans are characterized by changes in perception, cognition, emotions, and induction of psychotic-like symptoms that resemble early stages of schizophrenia (Vollenweider et al., 1998). Psilocybin also attenuates neurocognitive parameters in humans (e.g., disrupted sustained attention and altered visual information processing) and disrupts sensorimotor processing (Tyls et al., 2013), effects that are also typically disrupted in psychotic patients (Park and Holzman, 1992; Vollenweider et al., 1997, 1998; Geyer, 1998). Due to this phenomenological resemblance of acute intoxication with psychosis and its long history of safe clinical use (including psychiatric treatment) it is, nowadays, the most frequently used serotonergic model of psychosis in humans (Tyls et al., 2013). Furthermore, the therapeutic potential of psilocin/psilocybin has been recently investigated in several psychiatric disorders such as obsessive–compulsive disorder, anxiety, and addiction (Moreno et al., 2006; Grob et al., 2011; Bogenschutz and Pommy, 2012; Stebelska, 2013; dos Santos, 2014). Despite the evidence of how psilocybin changes cognitive performance in humans, little is known about its effects on cognition in animals.

To further investigate the effect of psilocin on cognition in animals we decided to test spatial memory using two spatial paradigms: the Morris water maze (MWM) and the Carousel maze. Spatial navigation as a behavioral manifestation of knowledge of the environment (i.e., cognitive map; Tolman, 1948) is considered as a useful and experimentally well-accessible animal analogy of declarative memory (O’Keefe and Nadel, 1978; Eichenbaum, 2001; Morris, 2013). Testing of spatial navigation requires sensitive spatial paradigms, such as the MWM (Morris, 1981; Morris et al., 1982; Stuchlik et al., 2007). This test can be used in many configurations, allowing testing of reference and working memory acquisition, consolidation, and retrieval. The water maze typically requires so-called allocentric spatial memory, i.e., guidance of navigation by spatial relationships of the distal visual cues located in the experimental room. Another powerful and relatively novel spatial paradigm is active place avoidance in a Carousel maze, previously also referred to as active allothetic place avoidance (AAPA) (Cimadevilla et al., 2000, 2001; Stuchlik et al., 2004; Wesierska et al., 2005). In this test, animals walking on a slowly rotating arena are required to avoid a sector located in a stable position of the room. It was demonstrated that rats avoiding places remember the arena- and room-based frames of reference (Fenton et al., 1998), which are continuously dissociated by the rotation of the arena. In its active place avoidance version, animals must, besides allocentric mapping, segregate spatial information into coherent representations of the room and arena frames and to select the room frame as the only one relevant for navigation. This ability was shown to strictly require hippocampus and termed cognitive coordination (Wesierska et al., 2005). Importantly, this task was demonstrated to require the presence of visual extra-arena information in the room frame (Fajnerova et al., 2014) as well as vestibular inertial stimulation caused by continuous arena rotation, which likely enhances the attention of the rats to distal visual cues (Blahna et al., 2011).

The aim of the present study is to test the hypothesis that psilocin attenuates acquisition of the Carousel maze (demanding mainly cognitive coordination) and reinforced retrieval and memory consolidation in the MWM (demanding allocentric memory). The effect of psilocin on the locomotor activity was assessed in the Carousel maze in order to dissociate effects upon cognition and/or motor and motivational functions.

## Materials and Methods

### Animals

All experimental procedures complied with the Animal Protection Code of the Czech Republic, the appropriate directive of the European Union (2010/63/EC) and NIH guidelines. Male adult Wistar rats (12–14 weeks, weighing 250–350 g) were obtained from the Institute’s accredited breeding colony. Animals were housed in pairs in 30 cm × 30 cm × 40 cm transparent plastic cages in a laboratory air-conditioned animal facility with a constant temperature (21°C) and 12:12 light/dark cycle with lights on at 7:00. Water and food were available *ad libitum* throughout the experiments. In all experiments, eight animals were used per group. Seventy-two animals were used in total.

### Drugs

Psilocin 1 and 4 mg/kg (synthesized at Pharmaceutical Faculty of Charles University in Prague, the structure was confirmed by mass spectroscopy and nuclear magnetic resonance techniques) was dissolved in saline (0.9% NaCl) acidified with 10 μl of glacial acetic acid. All injections were administered subcutaneously (s.c.) at a volume of 2 ml/kg. Fresh solutions were prepared every day, stored at 4°C and were protected from light.

### Experimental apparatuses and behavioral procedures

#### Carousel maze

The Carousel maze apparatus was described in detail in our previous study (Stuchlik et al., 2007, 2013). Briefly, it consisted of a smooth metallic circular arena (82 cm in diameter), enclosed with a 30-cm high transparent Plexiglas wall and elevated 1 m above the floor of a 4 m × 5 m room. The room contained an abundance of extra-maze landmarks. The rats were initially placed in the arena rotating at 1 rpm in a place directly opposite to the shock sector. Animals had to avoid a directly imperceptible 60° sector, defined in the North of the four arbitrary cardinal compass directions. The sector was identifiable solely by its relationships to distal room cues. A latex harness was attached between the shoulders of the rats, which carried an infrared light-emitting diode (LED). A computer-based tracking system (iTrack; Biosignal Group, USA) was located in an adjacent room. The tracking system recorded the rat’s position every 40 ms. Position time series were stored for off-line analyses (Track Analysis; Biosignal Group, USA). Whenever the rat entered the to-be-avoided sector for more than 0.5 s, the tracking system delivered a shock and counted an entrance. If the rat did not leave the sector, additional shocks were given every 1.4 s, but no more entrances were counted until the rat left the sector for more than 0.5 s. Mild shocks (50 Hz, 0.5 s, 0.4–0.7 mA) were administered from a computer-driven shock generator through the implanted low-impedance hypodermic needle implanted on the rats’ backs and through the contact between the rats’ keratinized paws and the grounded arena floor. Since the voltage drop is highest at the contact between rats’ paws and the floor, the rats “feel” the shock most likely in their feet. We avoided using a grid because in the Carousel maze it is necessary to allow accumulation of scent marks on the floor in order to generate a conflict between the arena and the room frames. The exact shock current, ranging between 0.4 and 0.7 mA, was adjusted for each rat to elicit a rapid escape response but not freezing. Rats were trained 4 days, 30 min after psilocin injection in four acquisition sessions of Carousel maze. On the fifth day, animals were tested without the presence of the drug (after-session). While the first four sessions evaluate the learning capacity, the last session would correspond to the short-term memory. The sector position was kept constant on the North compass direction throughout the training. The interval between sessions was 24 h. Eight animals were used in each group (24 rats in total).

#### Morris water maze

The MWM consisted of a blue-painted metallic circular tank (180 cm in diameter, 50 cm high) filled with water (20°C, 40 cm deep). A small, transparent Plexiglas escape platform (10 cm in diameter) was placed in the center of an arbitrarily defined Northeast (NE) quadrant of the pool and submerged 1.5 cm below the water surface. Rats were released facing the wall from the four cardinal compass directions (N, W, S, and E) in a quasi-random order. A trial stopped when the rat found the escape platform and climbed upon it. If the rat failed to find the escape platform in 60 s, the trial was stopped (recording latency of 60 s), and the rat was gently guided to the platform by the experimenter. The escape latency was recorded by the experimenter using a stopwatch. The rat was allowed to stay on the platform for 10 s and then it was placed to a waiting cage. One daily session consisted of eight swims (trials).

In the reinforced retrieval experiment four acquisition sessions (each consisting of eight swims) were conducted without the drug. On Day 5, psilocin was applied s.c. (1 or 4 mg/kg) 30 min prior to testing and the rats were released again for eight swims under the influence of the drug with the platform present in the same location of the pool (reinforced retrieval). Reinforced retrieval (actually re-acquisition) was chosen over a classical probe trial to also investigate re-acquisition under the drug. Five days later, drug-free animals underwent another re-acquisition with the platform present. Eight animals were used in each group (24 rats in total).

The experiment dedicated to testing the effect of psilocin on memory consolidation consisted of an initial 16 trials in the first session (Day 1) followed by psilocin injection immediately after the last swim at doses of 1 or 4 mg/kg, s.c. On the subsequent day (Day 2), the animals were released again for eight trials (swims) without any injections and the platform was again present in the same position (NE). Eight animals were used in each group (24 rats in total).

### Data analysis and statistics

The total distance traveled in a session (measured in the arena frame) reflected active locomotor activity without the contribution of the passive arena rotation. The distance was measured by the off-line tracking system by summing linear distances of points recorded each 1 s (the sampling frequency was 40 Hz). This sampling eliminated non-locomotor movements of the rat such as shivering. The number of entrances into the to-be-avoided sector (number of errors) measured the efficiency of avoidance in the Carousel maze. Another measure of the spatial performance within the session was the maximum time between the two entrances in a session (maximum time avoided). Results were analyzed using a two-way ANOVA (Groups × Sessions) with repeated measures on sessions, and Tukey’s HSD test was used when appropriate. Significance was accepted at *P* < 0.05.

In the MWM, we recorded the latency to find the platform as a measure of spatial memory (Morris, 1981; Stuchlik et al., 2007) and analyzed it with a two-way ANOVA (Groups × Sessions) with repeated measures on sessions. Tukey’s HSD test was used as *post hoc* when appropriate. We were not able to track the trajectories of the animals in this MWM experiment (however, locomotion was assessed in the Carousel maze; see the previous paragraph).

## Results

### Effect of psilocin on the acquisition of the Carousel maze

**Figure 1:**
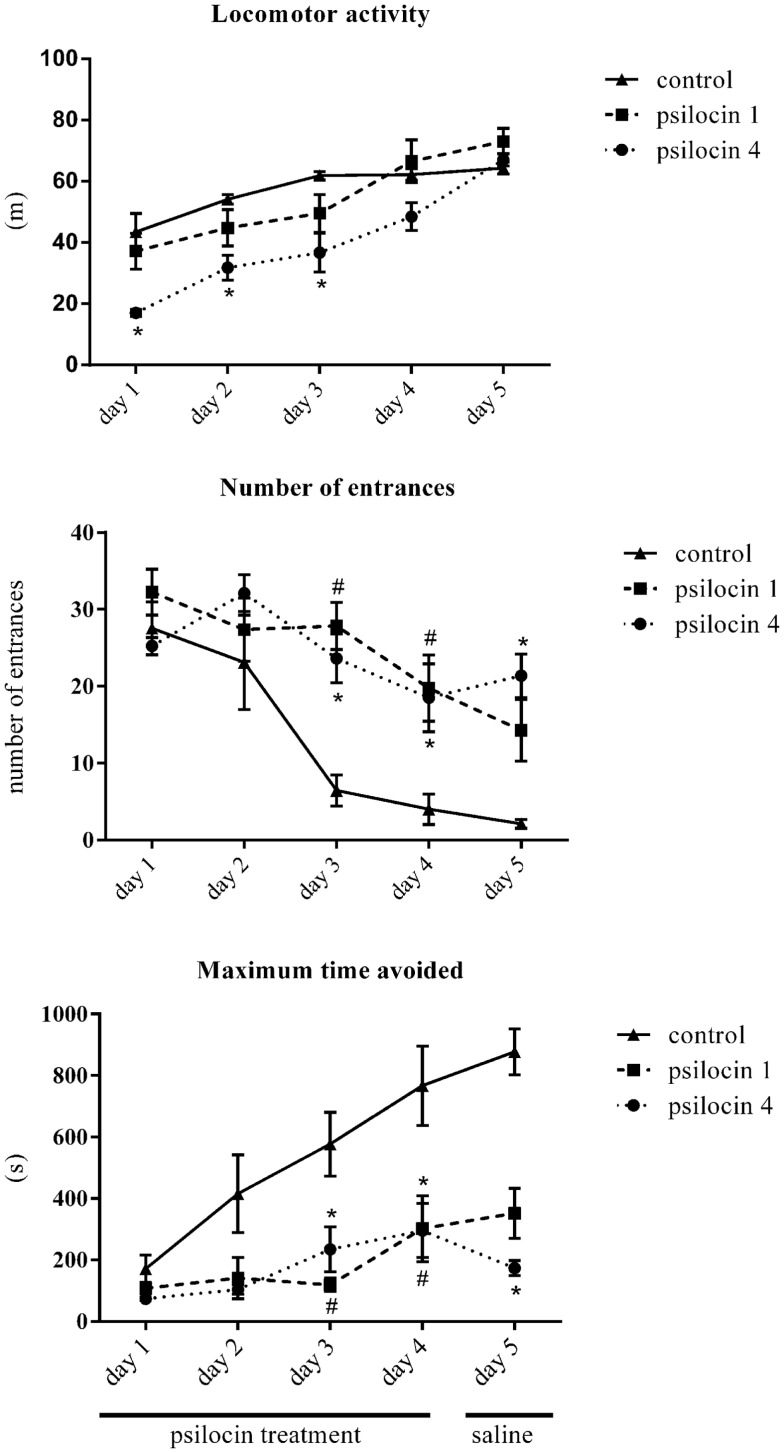
**The effect of psilocin on locomotor activity, number of entrances, and maximum time avoided in the Carousel maze**. Psilocin impaired spatial memory acquisition (higher number of entrances) at both doses. ^#^*P* < 0.05, psilocin 1 group (1 mg/kg, s.c.) compared to the control group on the same day. *P < 0.05, psilocin 4 group (4 mg/kg, s.c.) compared to the control group on the same day.

The animals did not exhibit any signs of stress or excessive discomfort during or after the drug injections. All data are summarized in Figure 1. First, we analyzed the locomotor activity, which was different between 1 mg/kg of psilocin, 4 mg/kg of psilocin, and the control group. A two-way ANOVA with repeated measures on sessions revealed a significant main effect of group [*F*(2, 22) = 8.51; *P* < 0.001] and sessions [*F*(4, 88) = 44.4; *P* < 0.0001] and interaction between the two factors [*F*(8, 88) = 3.47; *P* < 0.001]. The locomotor activity of the control group increased during the first three sessions and then stabilized to a constant level. There was no significant difference between the control group and the group treated with 1 mg/kg of psilocin (*P* > 0.05) during all five sessions. The group treated with 4 mg/kg of psilocin significantly differed from the control group only during the first three sessions (*P* < 0.05). These results show that a dose of 4 mg/kg of psilocin induced a decrease in locomotor activity but the locomotion of this group still increased with the training. This increasing trend persisted even in session 5 (the day without the application of psilocin), the locomotor activity was significantly higher in session 5 than in session 4 (*P* < 0.01). These results show that the dose of 4 mg/kg of psilocin impaired locomotor activity in the Carousel maze, whilst the dose of 1 mg/kg did not affect it.

The analysis of the number of entrances into the shock sector revealed differences between the studied groups. The two-way ANOVA showed a significant main effect of groups [*F*(2, 20) = 6.86; *P* < 0.01] and a main effect of sessions [*F*(4, 80) = 20.64; *P* < 0.0001]. There was also an effect of the interaction between these two factors [*F*(8, 80) = 3.95; *P* < 0.001]. A *post hoc* analysis of the factor of sessions showed that in the control group performance was better in sessions 3–5 compared to the first session (*P* < 0.05). The group treated with the low dose of psilocin (1 mg/kg) showed significant impairment in solving the Carousel maze task in sessions 3 and 4. The group treated with the higher dose of psilocin 4 mg/kg showed significant impairment in solving the Carousel maze in sessions 3 through 5. The higher dose of psilocin 4 mg/kg blocked the learning processes even in session 5 when the rats received saline instead of psilocin, although state-dependent learning might explain the impairment on the last day of training.

The maximum time avoided is generally strongly inversely correlated with the number of errors. Accordingly, analysis of this parameter also revealed differences between the studied groups. The two-way ANOVA showed a significant main effect of groups [*F*(2, 20) = 15.7; *P* < 0.0001] and a main effect of sessions [*F*(4, 80) = 12.5; *P* < 0.0001]. There was also an effect of the interaction between these two factors [*F*(8, 80) = 3.38; *P* < 0.01]. A *post hoc* comparison between the groups revealed that the control group exhibited a higher maximum time avoided in session 4 than the two groups treated with psilocin (1 and 4 mg/kg). There were no significant differences between the group treated with psilocin 1 and 4 mg/kg.

### Effect of psilocin on memory retrieval in the MWM

**Figure 2:**
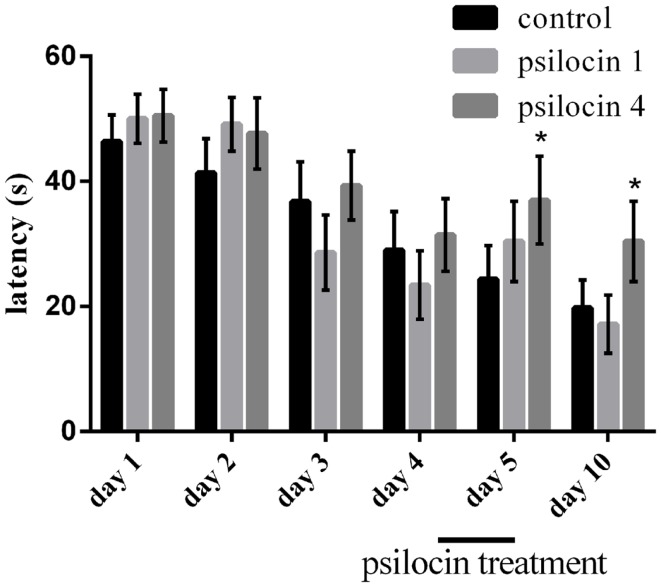
**Psilocin treatment (4 mg/kg) disrupted memory retrieval in the MWM after 4 days of pre-training and also impaired memory retrieval 5 days later (Day 10) without the presence of the drug**. *P < 0.05, compared to the control group on the same day.

We analyzed the behavioral parameter related to performance in the MWM, escape latency (Figure 2). Animals from all groups were pre-trained in the reference memory version of the MWM for 4 days without drug injections; the performance was similar across all of the groups [no significant effect between groups on Day 1–4 *F*(2, 20) = 2.1; *P* = 0.14]. There was a significant effect of days on all measures [*F*(2, 20) = 7.77; *P* < 0.0031]. On Day 5, memory retrieval was tested under the influence of psilocin (1 or 4 mg/kg, s.c). There was significant effect of days and sessions [*F*(2, 20) = 4.88; *P* < 0.01; *F*(7, 140) = 4.7; *P* < 0.0001]. *Post hoc* tests indicated that the retrieval was impaired in the group treated with a 4-mg/kg dose (*P* < 0.01). We found an effect of groups and sessions [*F*(2, 20) = 5.91; *P* < 0.01; *F*(7, 140) = 6.46; *P* < 0.0001] 5 days after the psilocin treatment. A control retrieval experiment was pursued without injections; surprisingly, the group with the previously administered high dose of psilocin was impaired again (*P* < 0.01).

### Effect of psilocin on memory consolidation in the MWM

**Figure 3:**
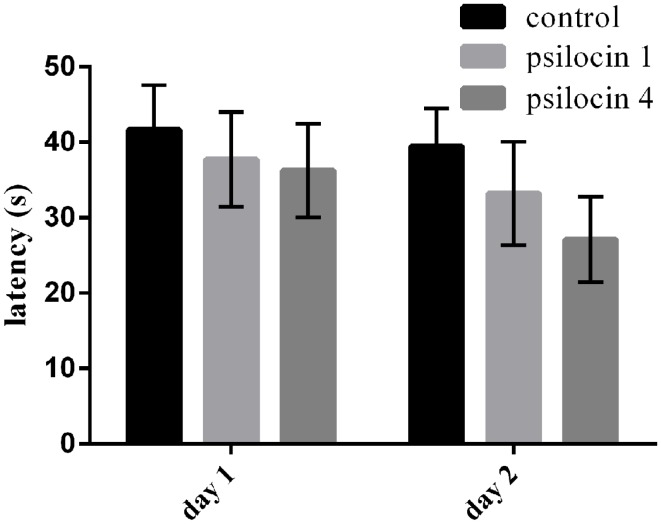
**Psilocin injected after training on Day 1 had no effect on memory consolidation at both doses (1 or 4 mg/kg) in the MWM**.

The animals were trained for 16 trials without drug injection on the first day (Day 1); subsequently, psilocin was injected (1 or 4 mg/kg) to investigate its effect of memory consolidation. On the next (Day 2), the animals were re-tested in the MWM with the same platform position. Psilocin did not affect the consolidation in the MWM. We found no effect of groups and sessions [*F*(2, 42) = 1.110; *P* = 0.3390; *F*(1, 42) = 1.163; *P* = 0.2870]. Figure 3 shows slightly better cognitive performance than on Day 1, but no significant between-group differences.

## Discussion

Our findings indicate that psilocin affects learning and memory acquisition necessary for spatial navigation and that it also has a disruptive effect on memory retrieval in MWM. Previous reports on the effects of psilocin/psilocybin on cognition in animals are very sparse, use different paradigms and did not bring uniform results, which make them difficult to compare. One study in rats reported impaired acquisition of a conditioned avoidance response (Sugrue, 1969) while another one in mice did not find any effect on the acquisition of an escape avoidance conditioning task (Collins et al., 1966). However, both studies were conducted with very high doses of psilocin/psilocybin. Furthermore, opposing effects of low psilocin doses on memory consolidation were found in two different mice strains when psilocin was injected immediately after each training session in a light–dark discrimination task (Castellano, 1978). On the other hand, most of the studies with other hallucinogens congruently found a disruption of cognitive performance. Similar findings to ours were shown for 2,5-dimethoxy-4-iodoamphetamine (DOI) in a special water maze, where this compound worsened the performance during acute intoxication as well as when subsequently tested without the influence of the drug (Kant et al., 1998). Early studies with LSD and mescaline congruently found disruption of spatial navigation, exploration, and discrimination within different frameworks in T-maze/Y-maze (Castellano, 1971; Davies and Redfern, 1973; Molinengo et al., 1986; Koupilova et al., 1989, 1999). LSD and other hallucinogens also induced errors in swimming through an underwater maze and increased starting latency of swimming through the maze (Uyeno, 1969, 1986). Recently, the use of a translational touch-screen based approach also showed alterations in visuo-spatial learning after acute LSD treatment in rats (Talpos et al., 2014). Finally, chronic mescaline treatment completely blocked the ability to switch between learned trials during operant conditioning (food reinforcement) (Fundaro et al., 1986).

There are two main aspects that have a crucial role in psilocin induced changes. Firstly, psilocybin, like other hallucinogens, had an inhibitory effect on locomotor and exploratory activity with the higher dose used (Collins et al., 1966; Halberstadt et al., 2011; Palenicek et al., 2012, 2013). However, we believe that the main effect of locomotor inhibition can be excluded, since the lower psilocin dose in the Carousel maze did not significantly differ from the controls during any of the sessions even though it induced the deficit in this task. Furthermore, the higher dose during session 4, when the animals showed peak performance, did not induce any significant locomotor inhibition. The psilocin-treated animals also showed a trend toward an increase in their locomotion within every session similarly to the control group, suggesting either developing tolerance to the drug or task sensitization. On the other hand, such an increase might also be attributable to the gradual acquisition of an avoidance strategy, which requires a highly coordinated spatiotemporal locomotion pattern in order to avoid a sector.

Secondly, since active avoidance in the Carousel maze as well as the MWM are spatial tasks requiring extra-maze cue orientation for successful acquisition, altered spatiotemporal perception (hallucinatory effects) can definitely contribute/underlie these changes. Unlike human studies on psilocybin (for a review, see Tyls et al., 2013); unfortunately, we are unable to directly confirm hallucinatory effects in rats in our setting. However, previous studies in rodents found an attenuation of time perception after hallucinogens (Hanks and González-Maeso, 2013) and studies in primates also support hallucinogenic effects in these species (Uyeno, 1969; Fantegrossi et al., 2004). Therefore, we might expect temporal and visual perceptual alterations to be present also in our setting. Indeed, it is well known that psilocin induces attention distraction (Hasler et al., 2004; Carter et al., 2005), which can also underlie the learning deficits. Interestingly, learning in the Carousal maze was also disrupted in the after-session, with no exposition to psilocin, indicating that psilocin blocked the learning process until the last session. Also, the higher dose of psilocin disrupted memory retrieval in the MWM, an effect that persisted for another 5 days. In other words, the rats that received psilocin during the retrieval trial did not improve their performance during this task, contrary to the control group, suggesting that not only retrieval but also re-acquisition was corrupted. This supports the idea that psilocin also had amnestic effects.

It is well known from the literature that psilocin effects are mainly mediated through 5-HT_2A/C_ and 5-HT_1A_ receptors (Tyls et al., 2013). These receptors are widely distributed in the neocortex, basal ganglia, limbic system, and hippocampus and in the case of 5-HT_1A_ also in rapheal nuclei (Barnes and Sharp, 1999). All of these areas are known to be involved in cognitive processes and memory. It is well known that 5-HT_2A/C_ receptors in particular are rapidly downregulated/desensitized after stimulation (Roth et al., 1990, 1995, 1998). This can explain the loss of the locomotor inhibitory effect observed in the Carousel maze. Since systemic administration of 5-HT_2A_ receptor antagonists induces cognitive deficit in rats (Ma and Yu, 1993; Fedotova and Ordyan, 2010) the down-regulation/desensitization of 5-HT_2A/C_ receptors after repeated administration of psilocin might also yield some of the effects observed in our setting. It is of interest that stimulation of 5-HT_1A_ and 5-HT_1B_ receptors is also known to impair acquisition of spatial memory tasks (Buhot et al., 1995; Herremans et al., 1995; Koenig et al., 2008). Nevertheless, further experiments are needed to explain the receptor mechanisms underlying these changes.

In conclusion, our study demonstrated that 5-HT_2A/C_ and 5-HT_1A_ agonist psilocin impaired spatial learning in the Carousel maze and it also had an amnestic effect in the MWM. On the other hand, it had no effect on memory consolidation.

## Conflict of Interest Statement

The authors declare that the research was conducted in the absence of any commercial or financial relationships that could be construed as a potential conflict of interest.
